# Alien Hand: Current Research Trends

**DOI:** 10.1007/s11910-025-01449-z

**Published:** 2025-09-19

**Authors:** Victor W. Mark

**Affiliations:** Departments of Physical Medicine and Rehabilitation, Neurology, and Psychology, University of Alabama at Birmingham, 619 19th Street South SRC 190, Birmingham, AL 35249-7330 USA

**Keywords:** Alien hand, Anarchic hand, Diagonistic dyspraxia, Corpus callosotomy, Split-brain

## Abstract

**Purpose of Review:**

This article reviews alien hand, a neurological disorder that causes involuntary limb movements that appear to be purposeful. This review outlines the history that identified the three widely accepted forms of alien hand and their distinct clinical and neuroimaging findings. This material summarizes behavioral disturbances that can occur with alien hand but are seldom addressed in a single review article, including propensity for self-injury, pathological sexual behavior, alien hand activities during sleep, and communication disturbances of alien Quality. The article then presents other paroxysmal, involuntary disturbances that are not usually considered to be alien hand, despite their appearing to be purposeful. Finally, this article reviews all PubMed-listed articles from 2020 to mid-2025 that addressed either alien hand or limb, or anarchic hand, for trends in understanding and treating this illness.

**Recent Findings:**

Meritorious advances in recent years included proposed checklists for the component behaviors for diagnosing alien hand, structured interviews for querying the patients’ experiences, and demonstrating white matter cerebral damage that extends far beyond the lesion boundaries that are identifiable on conventional structural brain MRI.

**Summary:**

This review summarizes the diversity of the presentations of alien hand. Many behaviors that are encompassed by alien hand are not so far explained by clinical or experimental brain MRI findings.

## Introduction

### Origins, History of the Nomenclature

After Paul Broca’s seminal 1860 s finding that associated left hemispheric structural brain lesion with acquired aphasia, late-19th century neuroclinicians became alert for other remarkable and disabling behavioral disorders that also could be traced to verified brain disease, such as unilateral spatial neglect [1] and acquired alexia [2]. At the turn of the century, a peculiar motor behavior was newly described, which involved limb movements that were incorrect for the task that the patient was asked to perform or during spontaneous self-care activities [3, 4]. For example, a patient could reach for an object involuntarily, intermittently fail to move the limb despite being asked or wanting to move it, or move a pencil to the mouth instead to a sheet of paper following instruction to write [3]. Involuntary mutually antagonistic actions between the patient’s hands were also noted, such as purposely dressing oneself with one hand and simultaneously and involuntarily undressing with the other [5]. These abnormalities were not due to spasticity, tremor, or incoordination, but instead erroneous object selection or movement sequences. The general disturbance was called “apraxia,” coined in 1871 [6]. However, these aberrancies were exceptional because only one arm was affected in most instances, while the patients could control the other hand. The patients did not understand the source for the hand’s abnormal movement, despite its appearance of purpose. It also seemed to them that the hand behaved as if it had a mind of its own, as if it had its own purpose. The disturbances generally upset the patients.

These behaviors initially were observed in persons with midline brain tumors. In the 1940 s similar disturbances were observed in patients who had undergone elective corpus callosotomy (“split-brain” surgery, which longitudinally divides the corpus callosum) to alleviate intractable seizures [7, 8]. A more specific term was introduced, “diagonistic dyspraxia,” to refer to the mutually opposing activities between the hands, in which one hand obeyed the patient’s aware intent, while the other performed the opposite action (e.g., dressing vs. undressing) [9]. Decades later, Brion and Jedynak noted that similar patients perceived an apparent foreign presence that affected one of the hands following the onset of brain tumors. This involved either the sense of lacking self-ownership of one’s hand when it was voluntarily grasped by the other hand out of view, or in one case, gibberish printing of words by the left hand that was in view, unlike the other hand’s writing. The latter patient considered that his aberrant writing was produced as if by another individual. From these observations Brion and Jedynak coined the term “le signe de la main étrangère,” or in English, the sign of the foreign hand [10] for these two different behaviors.

Later in the 1970 s the American neurosurgeon Bogen also observed unilateral seemingly purposeful hand behavior that was dissociated from conscious intent following corpus callosotomy for some epileptic patients. Because these behaviors resembled those that had been noted in the 1940s by Akelaitis et al., Bogen referred to this not only by Brion and Jedynak’s term “la main étrangère” but also, in English translation, “alien hand” [11]. In 1981 the term “alien hand” entered the peer-reviewed journal literature following the observations of apparently autonomous and purposeful behavior by one hand following cerebral infarction [12]. This term then became widely adopted, with more than 260 articles listed in the PubMed research journal article registry that have used this term.

Starting in the 1980 s, observations were published of involuntary levitation or other posturing of a solitary limb without the patient’s immediate attention to it, and the patient’s perception of its seeming to have a purpose, dissociated from the patient’s sensing any agency. This, too, was initially called “alien hand” [13]. Subsequently a journal report of this behavior termed this instead as “alien limb,” and this term was presented along with other previously described instances of “alien hand” [14]. “Alien limb” then also became popular, with now 198 articles that have used this term being listed in PubMed.

In 1991 the Italian investigators Della Sala and colleagues recommended the alternate term “anarchic hand” because the earlier “alien hand” description by Brion and Jedynak was ambiguous. Della Sala et al. advocated their term because the patients with involuntary, seemingly purposeful action by one hand nonetheless did not deny self-ownership of it [15]. As of now the PubMed registry lists 28 articles that have used the term “anarchic hand.”

However, despite the recommendation by Della Sala et al., “alien hand” is the term that remains more often used for seemingly purposeful behavior by one hand, not consciously instigated by the patient, and with the patient’s impression that an outside entity controlled the limb. Other terms have been used for the same general behavior, including “wayward hand,” “capricious hand,” and “Dr. Strangelove hand” (the last coined for the undesired, seemingly purposeful right hand actions by the title character of the 1964 Stanley Kubrick film). “Alien leg” or “alien foot” also have been rarely described. In many instances the terms “alien hand” and “alien limb” were used interchangeably in the same article. These two terms thus have become synonymous with each other.

### The Three Alien Hand Subtypes, and Related Subtypes

Although alien hand patients have shown diverse involuntary movements, three main disease subtypes have been proposed [16], which have conveniently organized these findings to facilitate identifying disabling behavioral patterns. These subtypes not only differ from each other clinically, but they are also associated with different anatomical areas of brain injury, located in different sites along the cerebral midline rostral-caudal axis. Feinberg et al. defined the first two in 1992 and recognized preliminary evidence for a third kind of alien hand [17].

i) The *frontal alien hand* involves disinhibited grasping and squeezing objects within reaching distance and usually in view. Involuntary gripping of bedclothes can also occur [18, 19]. Often it is difficult for the patient, and even the examiner, to free the hand from the object. The alien hand behavior can be so extreme as risking self-injury, for example in one instance by reaching for a burning napkin, which had to be restricted by the patient’s other hand [20]. Damage to the contralateral medial frontal cortex is most often associated.
ii) The *callosal alien hand* entails actions that are more complex than grasping or groping. This kind of alien hand can reverse actions voluntarily initiated with the other hand, such as turning the page of a book vs. closing the book by the other hand. Yet in other instances, the alien hand may perform a task unrelated to the actions done by the hand that was under cognizant control. For example, while one patient intentionally shaved himself, his other hand unzipped his jacket for no clear reason [21]. In addition, the hand may wildly misspell or omit letters when writing [5, 10, 15]. As with the frontal alien hand, the callosal alien hand can also risk self-injury, as for example the hand reaching for a hot pot while the patient is distracted by a different task [22] or the hand attempting to control the steering wheel in an erratic manner while the patient was safely controlling the car with the other hand [23]. As implied by the name, this form of hand follows injury to the corpus callosum or medial cortex adjacent to the callosum, generally farther back than the medial frontal cortex that is involved with frontal alien hand.
iii) *The posterior alien hand* refers to a range of involuntary limb movements of purposeful appearance that include a substantial deficit in somesthesis. Late in the 20th century, well after the other forms of alien hand had been described, Levine and Rinn proposed a third alien hand kind in 1986 when they observed a patient who was hospitalized for an acute posterior cerebral artery territory infarction [24]. The features included poor contralateral cutaneous sensation of the arm, its movements interrupted by pauses, marked ataxic movements during voluntary reaching, and self-aimed involuntary movements. These latter movements could be potentially injurious, including pinching her other arm and knocking off her eyeglasses. The movements were aggravated by emotional upset, reduced during physical rehabilitation. The patient perceived her movements as if controlled by another entity, and she talked to the hand to try to control its movements.

In 1998 Ay et al. observed similar behaviors in another patient who had posterior cerebral infarction, including self-injurious movements [25]. They termed this “sensory alien hand.” Bundick and Spinella in 2000 described yet another similar patient, following posterior cerebral artery infarction, who had spontaneous levitation of the arm [26]. They termed the illness “posterior alien hand.”

As of now, “posterior alien hand” or limb have been reported in 16 articles in PubMed, most recently in 2024. One instance of “PCA [posterior cerebral artery] alien limb syndrome” was also reported. In contrast, six publications on “sensory alien hand” or limb have appeared, two of which were comments on prior articles, the last one in 2012. Consequently, “posterior alien hand” or limb have become the preferred terms for this behavioral pattern.

iv) *Mixed*,* multivariant*,* or combined alien hand.* Many instances have been reported of multiple forms of alien hand within the same patient [27–37]. These findings thus demonstrate that although the various alien hand subtypes are characterized by cerebral injury in distinct different regions, in some cases focal cerebral injury can provoke multiple forms of alien hand, perhaps from more extensive injury.
v) *Bilateral alien hand* has been occasionally noticed despite intact limb control at other times in the patient [38–46].
vi) *Alien leg or foot* have been occasionally reported [45, 47–52]. These behaviors have included the discrepancy between the patient’s intended movement and the leg’s performance, involuntary foot tapping, or forcing the foot downward unwittingly while driving.

### Aspects of Alien Hand Behavior, Seldom Reviewed

The following observations are presented here because they are rarely addressed in reviews. They are pertinent to consider possible mechanisms for alien hand and can extend one’s awareness for the scope of the disorder.

i) *Self-injury*. Many studies have reported self-injury or risk of self-injury by the alien hand [15, 20, 24, 25, 48, 53–68]. Behaviors included self-slapping, choking, scratching the eye, reaching for scissors involuntarily, trying to drink from a too-hot cup, or battling for control of a steering wheel while driving.
ii) *Sleep.* Many remarkable reports have indicated alien hand activity while the patients were sleeping or preparing to sleep [26, 55–57, 60–62, 65, 70–74]. These activities included self-directed groping, slapping, or choking, roving movements under the bedclothes, or arm levitation. The actions often awoke the patients.
iii) *Sexual behavior*. Several case reports have indicated public self-scratching the genitals, lifting the skirt of a passerby, or touching a woman’s breasts or buttocks [15, 42, 67, 75–78]. The patients indicated that they had not willed these actions and were embarrassed.
iv) *Freezing of movement.* Unintended interruption or impaired initiation of limb movement have often been reported in alien hand [3, 21, 29, 34, 45, 47, 48, 65, 78, 79].
v) *Freezing of decision*. Persons with alien hand can show or complain with difficulty with decision making. Liepmann’s 1900 patient repeatedly picked up but then put down test blocks for no clear reason [5]. The case 2 patient reported by Akelaitis in 1944 showed the same behavior when attempting to buy bread [9]. Some patients complained of inability to decide between two opposing actions [52], even after resolution of intermanual conflict [80]. Another patient, who returned to work, found difficulty with decision making [81].
vi) *Alien communicative behavior*. In some instances, alien hand patients uttered incorrect words repeatedly, to their frustration, or contradicted themselves or other individuals in their speech or gestures. Case 1 studied by Akelaitis felt compelled to contradict everything that was said to her by her mother [9]. Gasquoine’s patient complained of the inability to suppress speaking aloud some of his thoughts [42]. The case 3 patient studied by Nishikawa et al. was frustrated by stating the opposite of what she had intended to say [65]. Two other patients frequently self-contradicted verbally after corpus callosal surgery [82, 83]. A split-brain patient, upon saying the incorrect the name of a presented color, shook his head, and then changed his answer [84]. A patient following bilateral paracallosal infarcts pointed to the incorrect answer on a facial recognition test, while the other hand pointed to the correct answer [67]. A patient with callosal trauma was plagued by “conflicting thoughts” [85]. After a callosal infarction, a patient denied having seen a stimulus in his left visual field, but at the same time nodded [86].

### Etiology and Epidemiology

Alien hand is reported most often following stroke or corticobasal degeneration [87]. One study found that alien hand could occur in 3% of patients with corpus callosum infarction [88]. Less often it has been noted to follow corpus callosum surgery, traumatic brain injury, subarachnoid hemorrhage, Creutzfeldt-Jakob disease, hereditary diffuse leukoencephalopathy, brain herniation, post-brain tumor excision, Parry-Romberg syndrome (progressive hemifacial atrophy), or Marchiafava-Bignami disease (cerebral atrophy from alcoholism). Even though corpus callosum agenesis, the congenital failure to develop the largest interhemispheric commissure, has only rarely been reported to occur with alien hand [48], one patient with corpus callosum agenesis was reported to have alien hand that resolved after discontinuing carbidopa/levodopa that had been prescribed to control tremor [89].

Alien hand is overwhelmingly described in adults. In a few instances alien hand has been reported in children [68, 90–92], as young as at age 9 years [93].

### Natural Course

Following acute brain injury, alien hand tends to appear after a delay of days to weeks afterward (e.g [80, 94]). However, in one case alien hand appeared within minutes after a cerebral infarction had developed during recovery from bronchoscopy [29]. In contrast, in a young child, seven years elapsed after a stroke before alien hand appeared [68]. Recovery after acute brain injury generally occurs under a year (e.g [34, 80, 95]). In one case the alien hand resolved within hours of onset after a stroke [96]. Less often, the disturbance can persist for years after brain injury (e.g [43, 51, 54, 97]). The natural course of alien hand that occurs in neurodegenerative disease, such as corticobasal syndrome or Alzheimer disease, is not discussed in the literature.

### Treatment

Owing to the rarity of alien hand, randomized treatment trials have not occurred. Treatment outcomes have instead been reported in single cases or small case series [16]. Conventional neurological rehabilitation efforts have been challenging and generally not successful [35, 98]. Mirror box training (where the patient looks at the mirror reflection of the good arm that practices motor tasks and superimposed over the impaired arm) has been associated with improvement [99], but without comparison to treatment as usual or other treatments. Redirecting attention from the hand that has repetitive grasping has had conflicting results [100, 101].

### Hypothesized Mechanisms for Alien Hand

The rare occurrence of alien hand has prevented developing and testing theories for its etiology. Based on (1) associated clinical findings and (2) basic laboratory primate research, different pathophysiological mechanisms for the three kinds of alien hand are generally recognized. *Frontal alien hand*, commonly resulting from medial frontal lobe injury, has been considered to result from the physiological disinhibition of the ipsilateral parietal cortex, which is oriented to explore the surrounding space [102]. *Callosal alien hand* is generally thought to emerge from the loss of interhemispheric neural communication following injury to the brain’s largest commissure, the corpus callosum [17]. Consequently, this injury can divide consciousness, in effect, producing two independently functioning units that each entails comprehensive mechanisms for planning and directing movement, perception, and contemplation [103]. These two separate units may thus combat for control of the body and the surrounding environment. However, in general, only one cerebral hemisphere, more often the left hemisphere, has substantial verbal expression ability and mediates inner speech [104]. As a result, the patient may be unable to express insight on the decision making that is done by the other, “silent” hemisphere. The observations of alien communicative behavior, referred to above, are consistent with this mechanism. *Posterior alien hand* is widely thought to result from the disruption of somatosensory processing of cutaneous input coming from the contralateral limb. With reduction of this input, the affected hand may drift in space involuntarily, causing levitation [57].

There remain many other aspects of alien hand that are not explained, including the occasional self-attack of the hand owner’s body, impulsive and even dangerous maneuvers (e.g., wrestling for control of the steering wheel between the hands while the patient is driving), and the sudden interruption of the course of limb movement or the inability to initiate movement, despite the patient’s verbalized intent for doing so. The posterior alien hand entails more than drifting of the limb without somatosensory input to the brain, and yet the complex actions in the posterior alien hand subtype are not readily explained by deficient cutaneous sensitivity.

### Cousins of Alien Hand

Alien hand stands out among the varieties of neurological symptoms and involuntary deficits that can follow brain illness in the awake individual. However, there is a large range of other involuntary, complex disturbances that can follow brain illness and resemble voluntary behavior. These also command clinical attention, but which are not referred to as alien hand. This may occur in part because the patients did not report the impression of an alternative entity taking control. There also has not been systematic evaluation for whether the patients had thought that they felt alienation. In some alien hand cases, alienation was also not described [61, 105]. These other disturbances are summarized in Table 1. Except for misoplegia, associated movement, and limb synkinesis, these behaviors reflect the environment’s apparently commandeering the patient’s actions, and in this manner similar to frontal and callosal alien hand.

**Table 1:** Summary of spontaneous behaviors following brain illness not reported as alien behavior

Behavior	Description	References
Misoplegia	Self-criticism and personification of an immobile limb, even physically punishing it	[106, 107]
Associated movement	Elbow flexion in an otherwise plegic arm, following yawning or cutaneous stimulation of the other arm	[108]
Limb synkinesis or synkinesia	Voluntary movement by one limb causes involuntary duplicated movement by another limb	[109]
Agonistic dyspraxia	Impaired hand involuntarily performs the action that was requested for the other hand	[110, 111]
Avoiding response	Fingers fan out or a limb withdraws upon presentation of a near stimulus	[40, 112–115]
Imitation or imitative behavior or echopraxia	Compulsive copying of another person’s action	[116]
Utilization behavior	Compulsive using a tool within reaching distance	[117]
Environmental dependency syndrome	Compulsive adopting an authoritative role appropriate for a professional or recreational setting, e.g., doctor’s office	[118–120]
Functional neurological disorder	Limb hyperkinesis or immobility provoked by formal examination or self-attention	[121]
Rooting	Turning the lips toward a stimulus when touched on the side of the mouth or presenting a visual stimulus near the face	[122]
Forced hyperphasia	Logorrhea prompted by a key word in conversation	[123]
Response-to-next-patient-stimulation	Automatically obeying instructions directed at a neighboring patient	[124]
Hyperlexia	Compulsive reading aloud material encountered in the environment	[125]

### Latest Progress in Alien Hand Research

This review summarizes research on the involuntary *actions* of the patients and not the failure to *perceive self-ownership* of one’s limb, the two behaviors that were recognized by Brion and Jedynak’s seminal research as part of the “sign of the foreign hand” [10]. This survey is directed at only actions because (1) current alien hand research emphasizes motor disturbances, rather than the somatosensory or perceptual impairments, and (2) these deficits are more likely to impair self-care. For research on the failure of bodily self-ownership, instead one can use the terms either “somatoparaphrenia” or “asomatognosia,” which are widely used [126]. The generally impaired awareness of one’s body or its parts can be formally assessed with a questionnaire to further research on impaired body self-ownership [127]. These efforts may help to disambiguate the term “alien hand.”

**Fig. 1:**
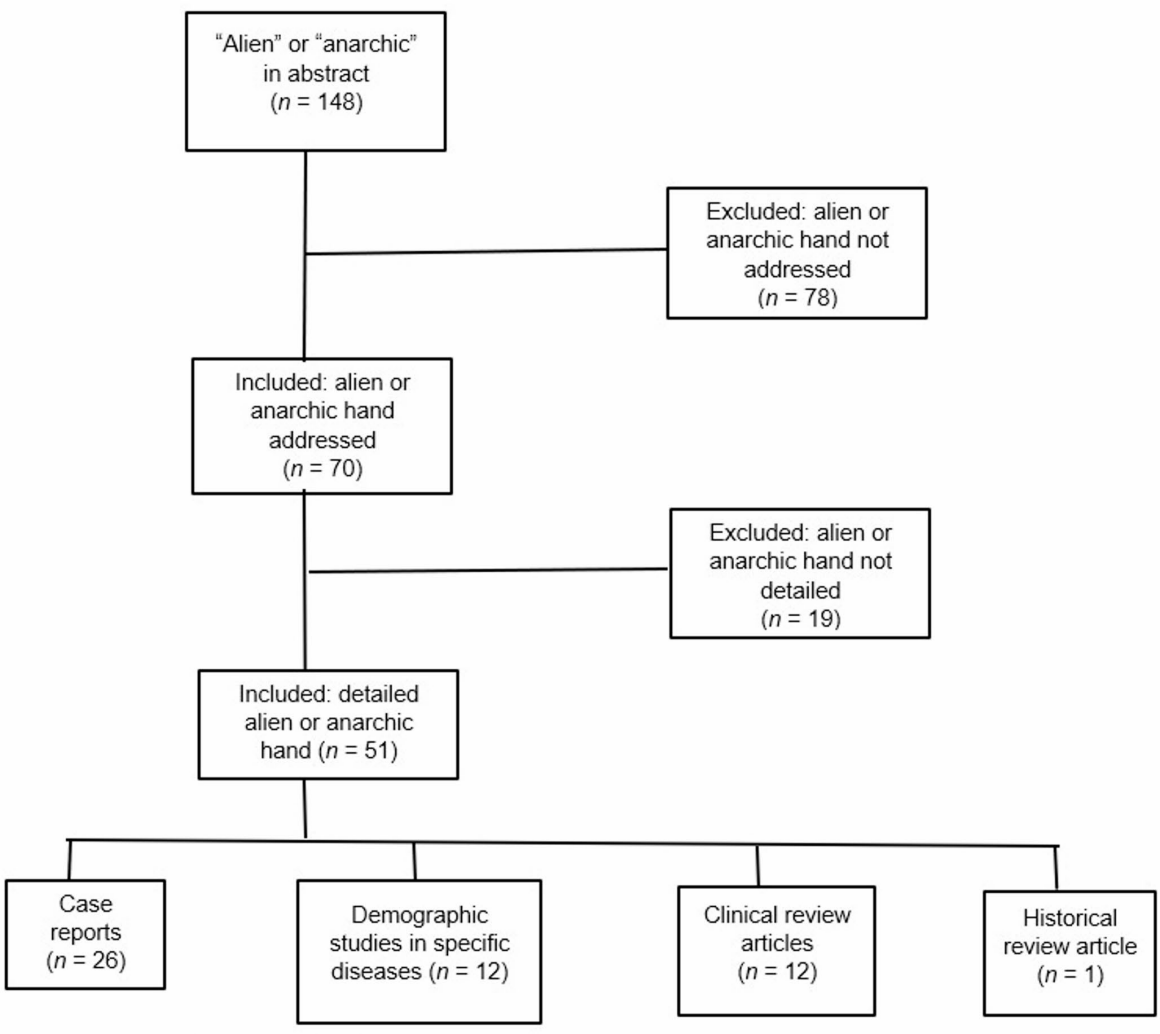
PubMed search results for articles on either “alien hand’” or “anarchic hand,” 2020–2025

The author searched PubMed for articles that were published from 2020 to July 2025 whose abstracts included “alien” or “anarchic,” in any language. This yielded 148 articles (Fig. 1). The articles were carefully reviewed. Articles that did not describe an alien neurological disorder of any sort (e.g., “alien species,” “alien particles,” “anarchic urbanization”) were removed, which left 70 articles. Inspecting these articles led to removing those that mentioned alien or anarchic hand without describing pertinent clinical phenomena; this left 51 articles. These remaining articles then were categorized as either case reports (*n* = 26), epidemiological studies of specific neurological disorders that included alien or anarchic hand (*n* = 12), clinical reviews of alien or anarchic hand (*n* = 12), or a historic review of alien hand (*n* = 1). Outcomes of the literature review are summarized below.

## Results

Many of the case reports or series did not appreciably add to the knowledge from the literature. Several articles also reported outcomes from different kinds of neurological rehabilitation or medication. However, because these were case reports of a rare illness, they did not establish treatment efficacy and were unable to conduct randomized controlled trials.

On the other hand, this period saw an increase in experimental neuroimaging studies in alien hand, in particular, the use of diffusion tensor imaging [36, 95, 128, 129]. These studies add to the previous few reports, which find that the white matter structural changes in alien hand extend considerably beyond the boundaries of the focal changes that are visible in standard structural brain MRI. An improved understanding of the extent of structural brain injury in alien hand could improve modeling how structural damage can cause this disorder. In a different approach, a study of alien hand in persons with corticobasal syndrome (*n* = 16) found atrophy in cortical areas that were physiologically connected to the precuneus (based on functional maps that had been developed from healthy control subjects), unlike corticobasal syndrome patients without alien hand (*n* = 25) [130]. These findings accord with a prior study of stroke patients with alien hand, whose lesions were also physiologically connected with the precuneus [131]. These findings suggest that the precuneus is physiologically involved with disturbances in sensing self-agency in individuals who have involuntary limb movement disorders.

Other notable findings or patterns from this period:

1. *Epidemiological studies*: From 157 patients with corpus callosum infarction evaluated over 11 years at a single site, five had alien hand, thus an incidence of 3% [88]. In a second series, among 15 patients who had undergone corpus callosotomy to control refractory seizures, three had persisting callosal alien hand [81]. These individuals were exceptional because they were left-handed, and clinical testing established that both of their hemispheres participated in language, with more language ability in the left hemisphere (test results were not provided). The alien hand in each case was on the left. The results suggest that the condition can persist because the right hemisphere lacks the controlling input from the left hemisphere’s language mechanism that would otherwise restrict the right hemisphere’s operating the contralateral hand. It does not appear that the right hemisphere’s language ability in these patients alone could override contralateral alien hand. This special situation may arise because the patient’s dominant hand was the left hand.
2. *Longitudinal studies*: Exceptionally long periods between the onset of alien hand and diagnosing the underlying disease have occurred in a couple of reports. In the first case, the patient’s sole presenting symptom was callosal alien hand, without abnormalities on brain imaging apart from corpus callosum thinning [132]. The alien hand symptoms then resolved. Further work-up was not implemented until two years later, when the patient developed memory impairment, parkinsonian signs, and advanced cerebral atrophy. Genetic testing confirmed adult-onset leukoencephalopathy with axonal spheroids. In the second case, the patient presented with a cerebral infarction in the territory of the right middle cerebral artery and unilateral spatial and motor neglect [133]. These disturbances resolved after two months, but which were replaced by intermanual conflict. The latter condition faded after four months. A third case was exceptional because alien hand preceded the onset of hemiparesis, rather than the opposite sequence, which more often occurs when hemiparesis is associated with alien hand [73].
3. *Developing a self-report assessment.* To facilitate identifying patients with alien hand, Lewis-Smith et al. assembled a 13-item inventory by which to query patients for occurrence of this condition [134]. This included asking about mirror limb movements, limb levitation, touching objects within reaching distance without intending, and impaired sensing self-agency of movements. This appears to be the first publication of a comprehensive self-report battery to screen participants for alien hand studies.
4. *A taxonomy for alien (or anarchic) hand behaviors.* Pacella et al. have proposed the distinguishing and distinct phenomena of alien hand (though they use the term “anarchic hand”): apparently purposeful movements, non-purposeful movements that can include limb levitation and self-grabbing, “uncontrolled” bilateral involuntary movements that can include intermanual conflict and diagonistic dyspraxia, miscellaneous behaviors that include lack of response to command, and personification of the hand or sense of an extracorporeal entity guiding the movements [95]. This system may benefit future alien hand research to improve comparing studies to each other.
5. *Unresolved developing a gold standard for diagnosing alien hand.* For some investigators, the term “alien hand” requires the patient’s inability to recognize self-ownership of the hand, regardless of the patterns of movement disturbance [63, 95]. This insistence stems from the original research by Brion and Jedynak [10], who found several patients’ failure to recognize self-ownership of the hand after brain injury and coined the term “la main étrangère,” which later became re-titled as “alien hand.” Nonetheless, other investigators use the term “alien hand” or “alien limb” to refer to apparently goal-oriented involuntary limb movement without referring to absent self-ownership of the hand (e.g [72, 128]). In addition, “alien hand” or “alien limb” are often reported without commenting whether the patient had complained of sensing an alien presence guiding the hand, while indicating the involuntary, apparent goal-directed limb behavior (e.g [80, 100, 132, 135]).
6. *Evidence for physiological interhemispheric disconnection after complete corpus callosotomy*. While not a study of alien hand and did not report this disturbance, a recent paper found that patients who had undergone complete corpus callosotomy had absent interhemispheric synchronization of resting fMRI activity [136]. This observation supports that following callosotomy, the patients’ cerebral hemispheres can function independently from each other. This in turn supports understanding callosal alien hand behavior: the hemisphere that operates the contralateral, alien hand is without inhibition from the other hemisphere, because callosotomy blocks interhemispheric neural communication. Conflicting of actions initiated by each hemisphere, independently from each other, can thus be disabling.

## Conclusions

The field of alien hand research is vibrant, with a steady output of about 12 peer-reviewed articles annually. This is remarkable for a rare neurological disorder. The illness continues to command neurological interest, which may lend insight for how impaired voluntary behavior can be caused by acquired brain injury and assist with managing this illness. However, the rare incidence of the illness impedes developing randomized controlled treatment trials. Consequently, treatment outcomes are anecdotal. Progress will remain slow.

Alien hand does not usually follow immediately after acute brain injury. Instead, usually several days or weeks elapse before the condition appears. This suggests that alien hand results from a slow, maladaptive physiological process during brain injury recovery, most likely because of neuroplastic cerebral reorganization. Such reorganization may well also support alien hand recovery under one year. Although the various forms of alien hand are associated with damage to specific brain regions, the rarity of the illness implies that lesion localization is not sufficient to account for its occurrence and characteristics. Of importance is that alien hand (1) only rarely occurs in children despite focal brain injury, and (2) almost never occurs in individuals with congenital corpus callosum agenesis. These observations suggest that brain development or maturation can influence alien hand.

Although alien hand is most often considered to be a disorder of active limb movement, many patients also demonstrate, paradoxically, paroxysmal motor or cognitive inactivity. The cause for this is not understood. Similarly, many other features of alien hand, summarized here, are not understood from reviewing the lesion characteristics or co-occurring signs, particularly self-injury, sexual behavior, occurrence during sleep, and communication disturbances. Another mystery is why alien hand is intermittent, while at other times the hand can comply with planned or requested movement. Still another unexplained aspect of callosal alien hand is the origination of diagonism (mutually opposite actions) and intermanual conflict (manual self-fighting to control self-care). These observations suggest that structural or physiological isolation of the cerebral hemispheres from each other can promote automatic oppositional or unwanted actions with the alien hand. This may not result from the “silent” hemisphere’s carefully and independently evaluating the patient’s needs, but rather from a reflexive state, in which the silent hemisphere impulsively reacts to the movements that are initiated by the other hemisphere and behaving at times inimical to the patient’s professed goals. The right cerebral hemisphere may be disposed to impulsive and rapid actions, as indicated by the several published observations that the alien left hand could launch into performing an action that was requested for the right hand before it could react [21, 52, 137]. This behavioral pattern accords with the many behaviors that are shown in Table 1, in which apparently autonomous manual behavior could be driven by features of the environment rather than careful decision making by the patient.

The large array of involuntary limb movements in Table 1 occurs without the patient’s reporting a foreign entity taking control of actions. Similarly, some alien hand case reports have not documented whether the patient had sensed alien control. These observations warrant systematic evaluation for whether these patients with behaviors in Table 1 truly consider these activities to be guided by an outside entity, or in contrast whether alien hand is a special condition in which the perception of self-agency can be dissociated from seemingly purposeful movements.

Different investigators prefer the terms “alien hand” vs. “anarchic hand” to describe seemingly purposeful limb actions for which the patient does not sense agency. Because “alien hand” is used more often, this term likely will remain the prevailing term.

## Key References

- Pacella V, Bertagnoli S, Danese R, Bulgarelli C, Gobbetto V, Ricciardi GK, et al. Anarchy in the brain: behavioural and neuroanatomical core of the anarchic hand syndrome. Cortex 2025;182:181-94. https://doi.org/10.1016/j.cortex.2024.10.017.
  - Identifies five key behavioral patterns of alien or anarchic hand.
- Lewis-Smith DJ, Wolpe N, Ghosh BCP, Rowe JB. Alien limb in the corticobasal syndrome: phenomenological characteristics and relationship to apraxia. J Neurol 2020;267:1147-1157. https://doi.org/10.1007/s00415-019-09672-8.
  - Provides a checklist of behaviors that distinguish alien hand, to be used when querying patients who may have the disorder, to support investigation.
- Santander T, Bekir S, Paul T, et al. Full inter-hemispheric integration sustained by a fraction of posterior callosal fibers. bioRxiv 2025. https://doi.org/10.1101/2025.02.14.638327.
  - Quantitative neuroimaging study of patients following corpus callosotomy, demonstrating physiological isolation of the cerebral hemispheres from each other.

## Data Availability

No datasets were generated or analysed during the current study.
